# The role of non-thermal atmospheric pressure biocompatible plasma in the differentiation of osteoblastic precursor cells, MC3T3-E1

**DOI:** 10.18632/oncotarget.16821

**Published:** 2017-04-04

**Authors:** Ihn Han, Eun Ha Choi

**Affiliations:** ^1^ Plasma Bioscience Research Center, Kwangwoon University, Seoul 01897, Korea; ^2^ Department of Electrical and Biological Physics, Kwangwoon University, Seoul 01897, Korea

**Keywords:** non-thermal atmospheric pressure plasma, non-thermal biocompatible plasma, osteogenic differentiation, FOXO1 signaling, osteoblastic cell lines

## Abstract

Non-thermal atmospheric pressure plasma is ionized matter, composed of highly reactive species that include positive ions, negative ions, free radicals, neutral atoms, and molecules. Recent reports have suggested that non-thermal biocompatible plasma (NBP) can selectively kill a variety of cancer cells, and promote stem cell differentiation. However as of yet, the regulation of proliferation and differentiation potential of NBP has been poorly understood.

Here, we investigated the effects of NBP on the osteogenic differentiation of precursor cell lines of osteoblasts, MC3T3 E1 and SaOS-2. For *in vitro* osteogenic differentiation, precursor cell lines were treated with NBP, and cultured with osteogenic induction medium. After 10 days of treatment, the NBP was shown to be effective in osteogenic differentiation in MC3T3 E1 cells by von Kossa and Alizarin Red S staining assay. Real-time PCR was then performed to investigate the expression of osteogenic specific genes, *Runx2*, *OCN*, *COL1*, *ALP* and *osterix* in MC3T3 E1 cells after treatment with NBP for 4 days. Furthermore, analysis of the protein expression showed that NBP treatment significantly reduced PI3K/AKT signaling and MAPK family signaling. However, p38 controlled phosphorylation of transcription factor forkhead box O1 (FoxO1) that related to cell differentiation with increased phosphorylated p38. These results suggest that non-thermal atmospheric pressure plasma can induce osteogenic differentiation, and enhance bone formation.

## INTRODUCTION

Plasma is a fourth state of matter, after liquid, solid, and gas. In nature, plasma is widespread in outer space. Plasma created in an enclosed vacuum chamber using radio frequency or microwave energy is generated charged particles. One of the technologies for medical device sterilization termed NBP has been in use since the 1990s. The advantages of NBP sterilization are that it provides safe, non-toxic, dry, and low-temperature sterilization. After sterilization, the charged particles form free radicals, which combine to form water and oxygen. When applied, free radicals evaporate, spread, and successfully kill bacteria, viruses and fungi on all surfaces they can reach. Recently, NBP shows anti-cancer effect in a variety of cancers from brain, breast, prostate, ovary and lung [1–8]. Furthermore, several devices have been reported using plasma needle for medical application [9–12].

To date, the osteogenic ability of bone formation mainly depends on different cell sources or specific cytokines, such as growth factors, and hormones. For tissue engineering, advanced methods can be designed in three-dimensional culture condition with specific physical [13–15] and mechanical co-factors [16–18]. The development of a differentiation method is also important to successful biomedical engineering and application. Recently, it reported that NBP may be responsible for differentiation and proliferation on stem cells cause ROS generation by NBP. However, there is little evidence with NBP treatment for stem cell differentiation [19].

In this study, we evaluated the effects of NBP on MC3T3-E1 and SaOS-2 cell lines on osteogenic differentiation. We also investigated the selectivity and feasibility of NBP only for the differentiation of osteoblastic cells, and for medical application.

## RESULTS

### NBP inhibited proliferation of osteoblastic cell lines *in vitro*

After 24 hours treatment, the number of decreased MC3T3-E1 cells was dependent on the treatment time of NBP. But 5 days later, these cells were growing more than control group. NBP affected SaOS-2 cells less than MC3T3-E1 cells. However, SaOS-2 cells showed decreased proliferative rate after 5 days (Figure 2).

**Figure 1:**
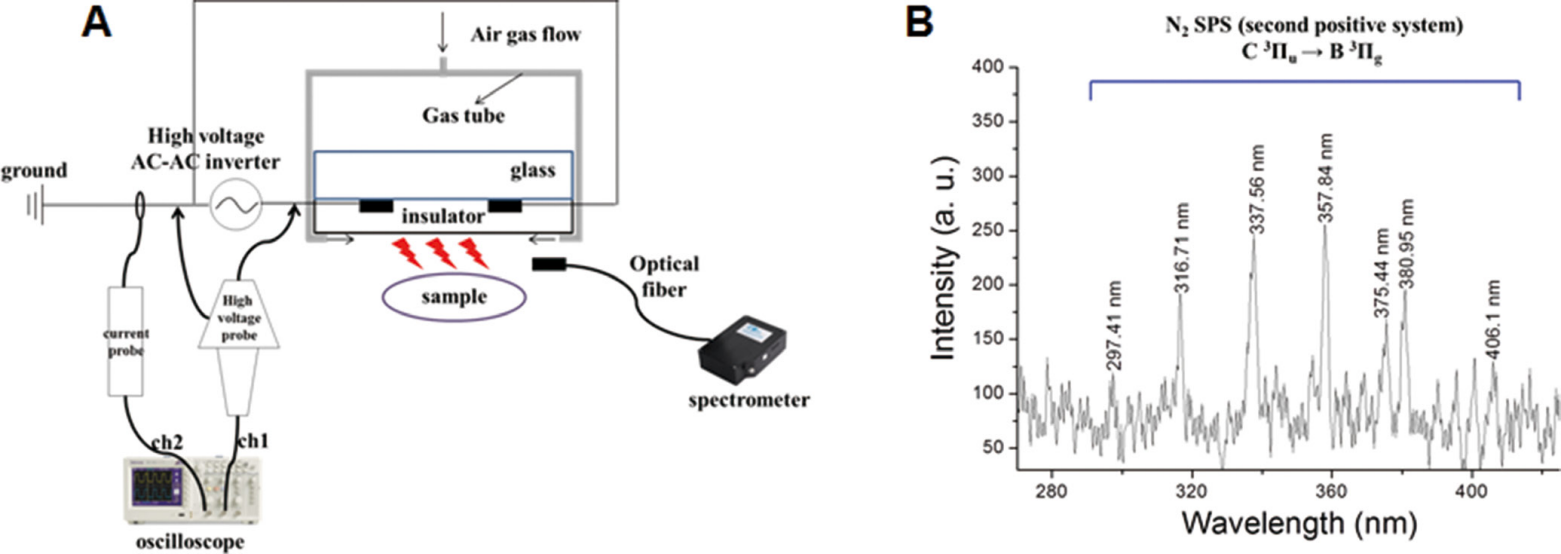
Schematic illustration of NBP device and OES spectrum

**Figure 2:**
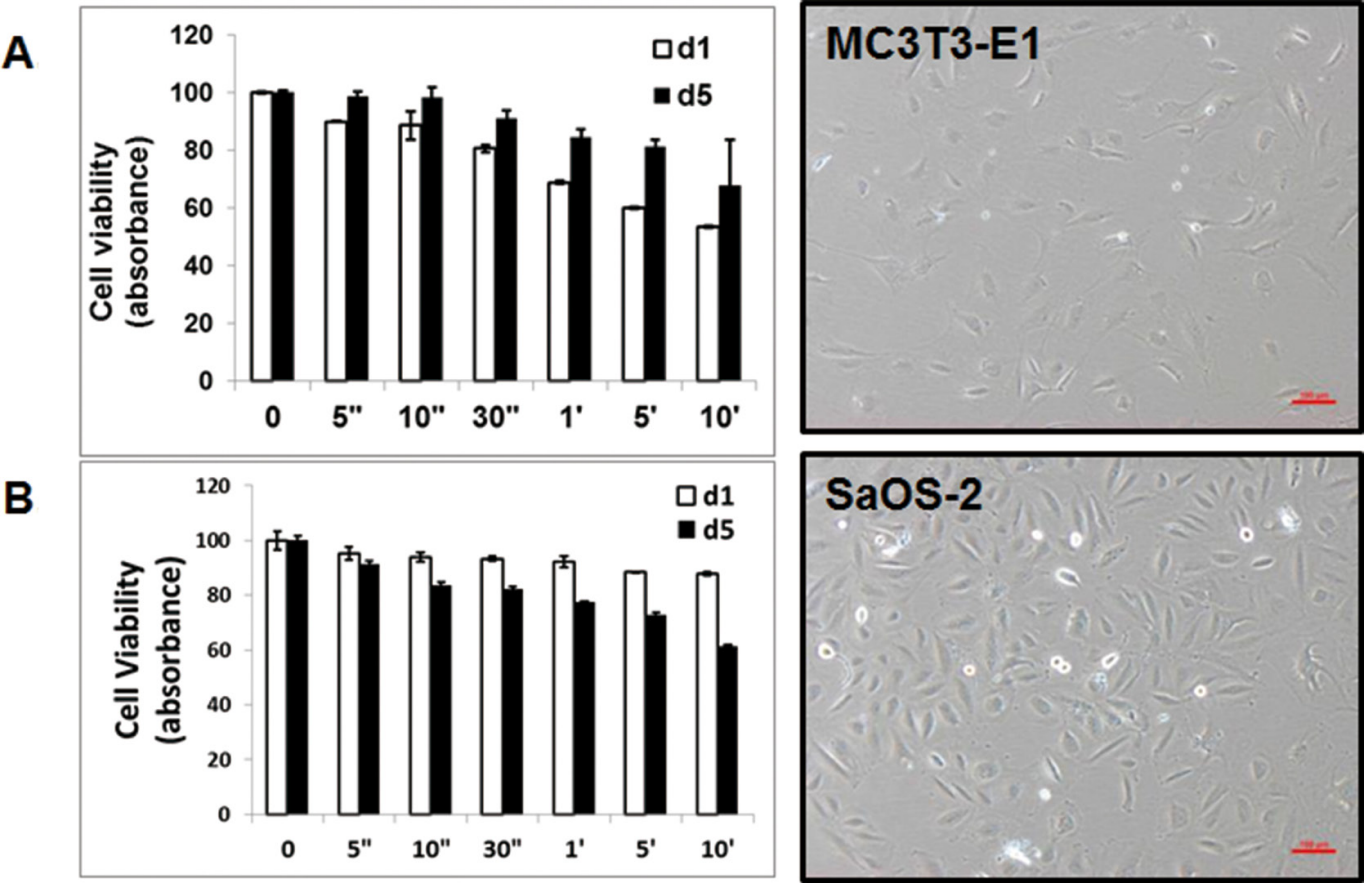
Morphology and cytotoxicity by atmospheric-pressure plasma (**A**) MC3T3-E1 cells and SaOS-2 (**B**) were cultured without plasma treatment. Cytotoxicity assay was performed with CCK-8 solution for day 1 and day 5 after plasma treatment.

### Effects of NBP on the apoptosis of osteoblastic cell lines

**Figure 3:**
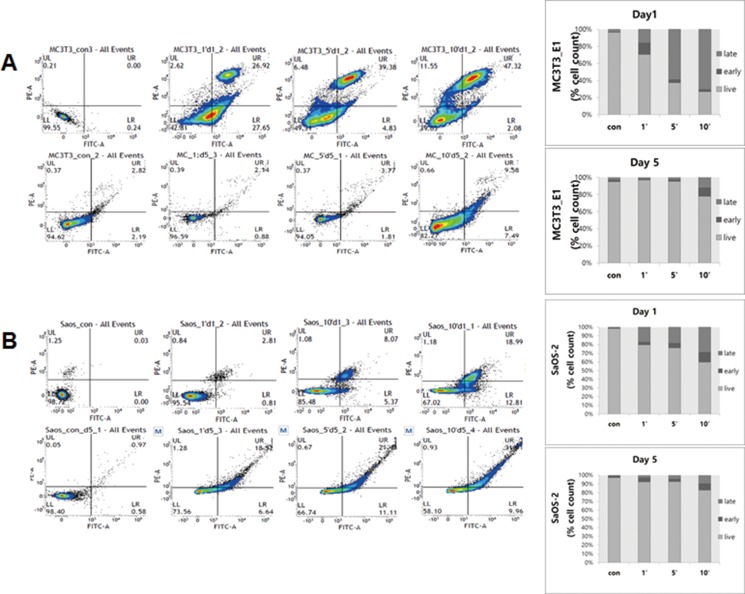
Apoptosis of osteoblast cells, MC3T3-E1 (**A**) and SaOS-2 (**B**) on day 1 (top) and day 5 (bottom) after plasma treatment for Annexin V-FITC assay. Apoptotic cells were evaluated by flow cytometer.

We evaluated the apoptosis of MC3T3-E1 and SaOS-2 cells using flow cytometry incubated cells for 1 day and 5 days after plasma treatment. After 1 day, the apoptotic ratio of MC3T3-E1 was higher in the 10 min group (early apoptosis of 2.29 ± 0.85% and late apoptosis of 59.7 ± 19.52%) compared to SaOS-2 in the 10 min group (early apoptosis of 7.1 ± 4.14% and late apoptosis of 9.82 ± 6.88%) (Figure 3A). After 5 days, the percentage of SaOS-2 cells in the apoptosis phase increased to 11.28 ± 0.98% of early apoptosis and 28.69 ± 1.69% of late apoptosis, as compared to 9.76 ± 1.84% of early apoptosis and 12.18 ± 4.41% of late apoptosis in the MC3T3-E1 cells (Figure 3B).

### Effects of NBP on osteogenic differentiation of osteoblastic cell lines

**Figure 4:**
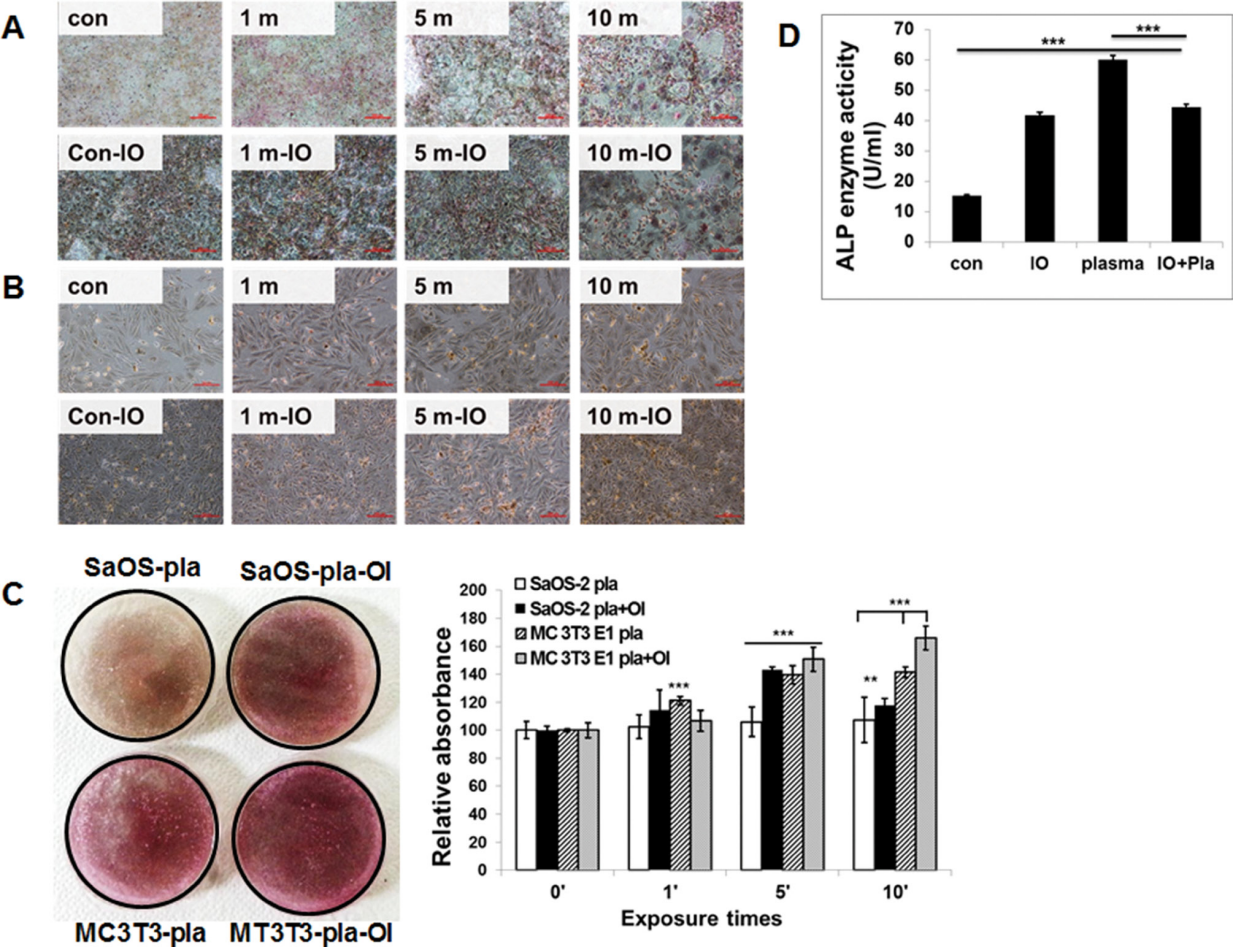
Effect of NBP on the mineralization of extracellular matrix by MC3T3-E1 cells (**A**) and SaOS-1 cells (**B**). Osteoblastic cells were exposed to NBP for various time with or without OI medium and von Kossa staining (A and B) was performed to demonstrate mineralized nodule formation at days 10. Both the SaOS-2 plasma and MC3T3-E1 plasma combined with OI media groups showed significant differences by one-way ANOVA that group F_(7.581)_ = 0.655, *p* = 0.0042, *p* < 0.01. Quantitative analysis of ARS performed by determine absorbance at 450 nm wavelength (**C**). MC3T3-E1 cells were treated with NBP with or without OI medium and ALP activity was measured by absorbance at 405 nm (**D**). Compared all groups showed significant differences by one-way ANOVA: group F_(313.8)_ = 0.9874, *P* < 0.0001 But there is no difference between plasma and plasma with OI group.

We investigated the effect of osteogenic differentiation by NBP. The stimulatory effect of NBP was comparable to that of osteogenic differentiation medium only. When the cultures were simultaneously treated to cold plasma and osteogenic induction medium, osteogenic activity was enhanced in both MC3T3-E1 and SaOS-2 cells. The stimulatory effect was also observed in treatment group of cold plasma only (Figure 4). The NBP treated for 5 min in MC3T3-E1 cells (Figure 4A) and for 10 min in SaOS-2 cells (Figure 4B) showed more stained cells by von Kossa staining. Also, mineral nodule formation with Alizarin Red S staining confirmed the differentiation of osteogenic cells. We obtained similar results with von Kossa assay that treatment with NBP enhanced mineralized materials (Figure 4C). The MC3T3-E1 cells showed morphological change 10 days after NBP treatment with or without osteogenic induction. Additionally, we evaluated ALP activity in the cell secreted factors after plasma treatment. The ALP activity showed a significant increase in the plasma treatment group compared with controls. These results indicate that NBP can stimulate osteogenic differentiation.

### NBP stimulates differentiation genes of osteoblastic cells

**Figure 5:**
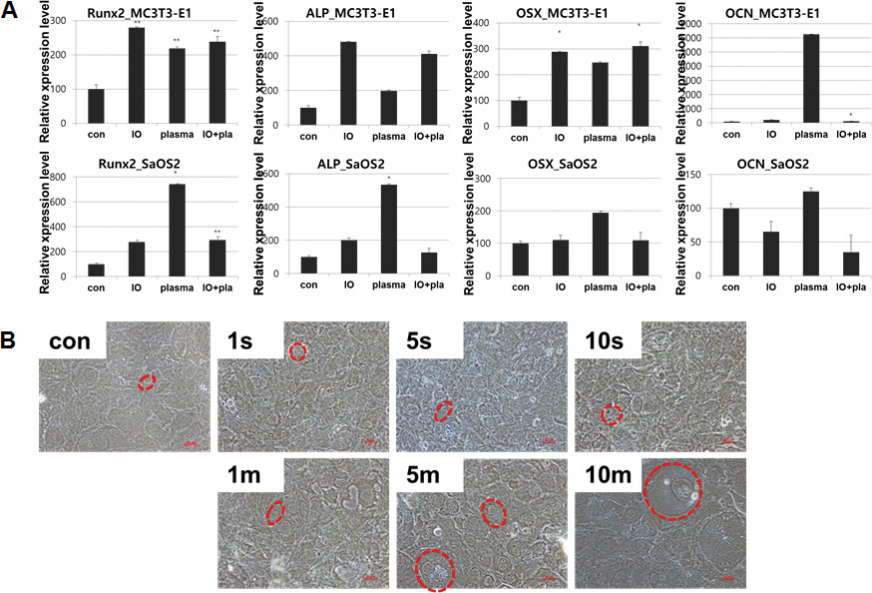
(**A**) Gene expression after treatment of NBP. (**B**) Morphological change of osteoblast cells cultured for 10 days after plasma treatment w/o osteogenic induction. Cells were visualized under microscope (magnification; 20×).

MC3T3-E1 and SaOS-2 were assessed for the expression of osteogenic genes at cold plasma treatment for 5 min with or without osteogenic medium (Figure 5). Of all the genes of interest, *OCN* and *Runx2* were expressed after 4 days from NBP treatment without osteogenic induction. Like *Runx2*, *ALP*, *OCN*, and *osterix*, this osteogenic marker was increased by NBP compared with control group, even though it was less than for the osteogenic induction group. Also, *Runx2*, *ALP*, and *osterix* genes were more highly expressed in SaOS-2 cells in the NBP only group than in the osteogenic induction group. After treatment with NBP, MC3T3-E1 cells showed morphological change by treatment time of NBP that comprised gradual increase in size, and having few arms, like the typical osteocyte cell shape.

### Effects of NBP on FoxO1 mediates pathway of osteoblastic cells

**Figure 6:**
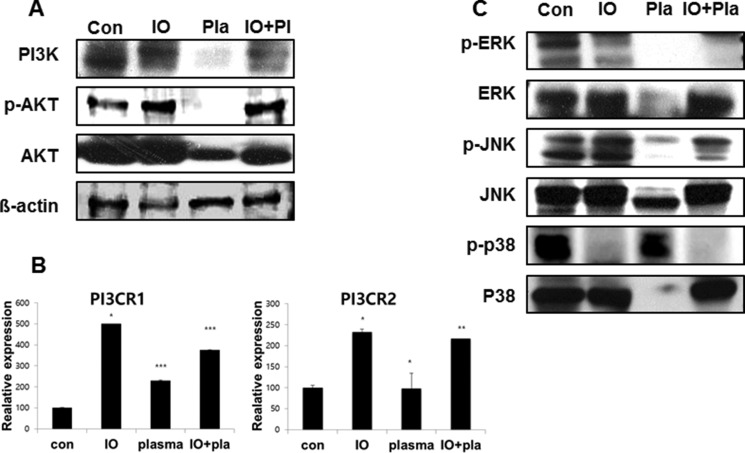
Expression of PI3K in MC3T3-E1 cells (**A**) Western blotting was performed to determine the expression of PI3K and AKT. (**B**) qReal-time PCR was performed with PIK3R1 (p85-a), PIK3R2 (p85-b); regulatory subunits, primer as PI3CA (p110-a; catalytic) subunits. MAPK family assessed by immunoblot for ERK, JNK and p38 (**C**).

Cell lysates were prepared from MC3T3-E1 cells treated with plasma for 5 min, and subjected to Western blot analysis. Figure 6A shows that NBP treated MC3T3-E1 cells revealed decreased expression of PI3K and AKT compared with the non-treated control group. NBP decreased those PI3CR1 and PI3CR2 gene expression levels compared with the osteogenic induction group, which indicated the same result of protein expression level as that of the NBP treated group (Figure 6B). We then performed Western blotting to detect whether MAPK family are involved in the regulation of cell proliferation and differentiation. The level of phospho-p38 MAPK in the plasma treated group was higher than in normal (Figure 6C), whereas plasma treatment markedly decreased the level of phospho-JNK and phosphor-ERK, which was associated with decreased phosphorylated AKT.

**Figure 7:**
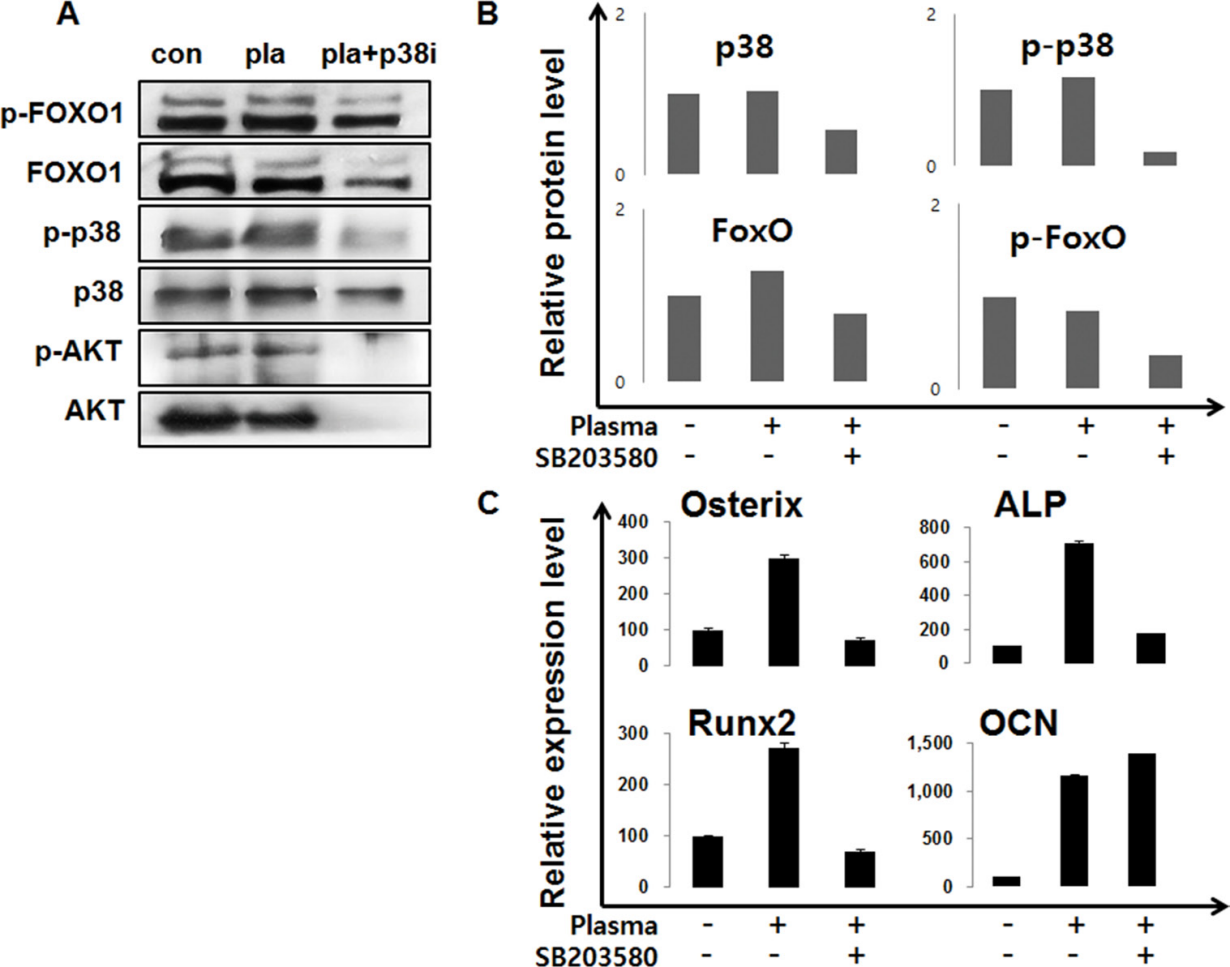
MC3T3-E1 cells were treated with NBP and SB203580 and performed by immunoblot to evaluated FoxO1 and AKT expression (**A**). The cells were treated and collected for real-time qPCR using osterix, ALP, Runx2 and OCN (**B**).

**Figure 8:**
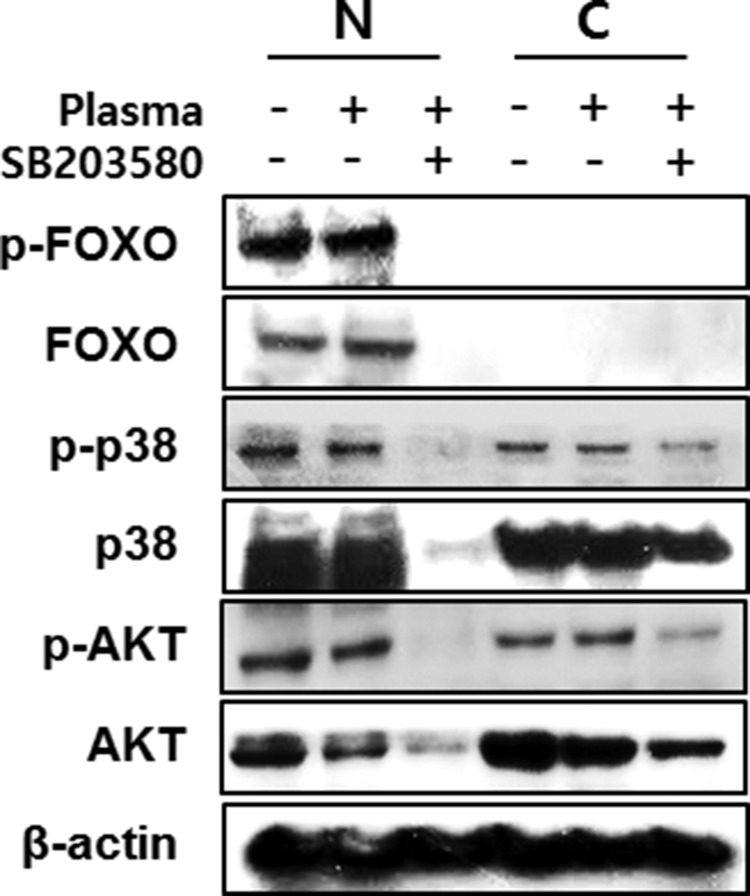
MC3T3-E1 cells were treated with NBP and SB203580 and separated nucleus and cytosol fraction after harvested the protein The cells were assessed with FoxO1, p38 and AKT by immunoblot.

We investigated the FoxO1 role in osteoblastic cells after treatment of NBP. The levels of FOXO1 protein levels did not show significant difference between the non-treated and NBP treated groups. However, after treatment with p38 inhibitor SB203580, plasma treatment decreased the FoxO1 and phosphorylated FoxO1. (Figure 7A and 7B). The same result was also shown in AKT that decreased with the NBP treated group, and disappeared after treatment together with SB203580. After NBP treatment of MC3T3-E1 cells, osteogenic gene expression compared with control group increased, such as osterix, ALP, Runx2 and OCN, and then those gene expressions treated with SB203580 decreased (Figure 7C). We evaluated FoxO1 and p38 by NBP application. When used with SB203580, p38 obviously disappeared in the cytoplasm and nucleus, which lead to decreased FoxO1 expression and AKT expression level after treatment with SB203580 (Figure 8).

## DISCUSSION

Cancer therapy using NBP is an emerging area of medical convergence. In this study, we found that both MC3T3-E1 and SaOS2 cells showed osteogenic differentiation by NBP treatment. Both MC3T3-E1 and SaOS-2 cells are well characterized cell lines, derived from osteoblast of normal calvaria and osteosarcoma respectively. MC3T3-E1 cells showed decreased cell viability 24 hours after NBP treatment. Only 53.5 ± 0.23% of cells survived in the 10 min treated group, whereas 67.9 ± 15.8% of cells remained viable 5 days after treatment of NBP. In contrast, SaOS-2 showed viable in 87.8 ± 0.63% of cells after 10 min treatment, but after 5 days consecutive influence of NBP, decreased to 61.5 ± 0.4% (Figure 1). We consider that these differences can explain cancer cell specific suppression of NBPs. In this regard, we have shown that the results of apoptosis revealed similar patterns (Figure 2). Previous reports show that endogenous RONS generated from NBP provoke more hyperactivated metabolism in cancer cells than normal cells [20].

Both cells showed mineral nodule formation in Von Kossa staining without osteogenic induction after 5 min NBP application. Interestingly, after 5 min NBP treatment, MC3T3-E1 cells changed their shape. Morphological changes of cells were found in round and large shapes that resemble osteocyte.

ALP is known as an early marker of osteogenic differentiation. A recent study demonstrates the ability of NBP to elevate ALP activity as a new efficacious strategy to influence human MSCs [21]. The results of ALP activity indicated the significant effect of NBP on ALP activity, as compared to control cells, and even osteogenic induced cells (Figure 2D). This finding suggests that NBP can stimulate osteogenic differentiation faster than can osteogenic induction molecules.

We found that phosphorylation of FOXO1 gene was related to modification of protein expression. FOXO is a transcription factor that belongs to the forkhead gene family. This molecule is found in mammal, and is composed of subfamilies, such as FOXO1, FOXO3a, FOXO4, and FOXO6. In many studies, FOXO is known to be an important regulator of cell cycle arrest, DNA repair, proliferation, apoptosis, and cellular metabolism to oxidative-stress. This molecule reacts with insulin and growth factors, is phosphorylated by PI3 kinase/Akt pathway, combines with 14-3-3, and finally dissolves, after translocation from nucleus to cytoplasm. Without insulin and growth factors, dephosphorylation of FOXO leads to movement of FOXO to nucleus, then transcription of related genes [22]. SIRT1 can inhibit FOXO function by acetylation, and oxidative stress provokes JNK and MST mediated FOXO phosphorylation and subsequent gene transcription [23–25]. FOXO is also phosphorylated by ERK and p38 MAPK component, which are related to extracellular signal transduction [26, 27]. MAPK-induced FOXO1 phosphorylation stimulates VEGFR2 transcription, which is a principal factor of neovascularization. In this study, NBP treated MC3T3-E1 cells stimulate FOXO1, which was not caused by the suppression of PI3K/AKT signaling pathway. Subsequent FOXO1 can result in differentiation of osteoblastic cells, as previous reports have shown [28]. p-38 phosphorylation lead to activation of FOXO1, which might suppress ROS removals. Finally, p38-mediated expression stimulates the osteogenic genes, such as *Runx2* and *ALP*, and differentiation of osteoblast to osteocyte by OCN and osterix. Although osteogenic induction by growth factors and cytokines might be the most effective way to osteogenesis, NBP may be useful in clinical applications.

### Graphic abstract

Under normal condition, intracellular PI3K/AKT pathway inhibits FoxO related gene transcription. However, NBP treatment can generates RONS that block PI3K/AKT pathway and increases p38 signaling to stimulates osteogenic differentiation.

## MATERIALS AND METHODS

### Plasma device and treatment

Figure 1A shows a schematic of the micro dielectric barrier discharge (DBD) NBP device used in this study, with the electrode thickness 5 μm, electrode gap 200 μm, dielectric layer of glass thickness 30 μm, and insulator layer 1 μm. The electric power for NBP generation was used by alternating current (AC) power supply (15.4 kHz), with a high voltage AC-AC inverter (PNP-1000, Daekwang Electric Co., Seoul, Korea). Plasma was discharged with a breakdown voltage of 500 V and breakdown electric current of 13 mA. Nitrogen gas (N_2_) with a flow rate of 1.5 liters per minute (lpm) and 25 millisecond (ms) on-time was used for NBP generation. Current and voltage profiles during micro DBD were acquired using a magnetic pickup coil and 1000× voltage probe, respectively, with an oscilloscope (Tektronix, Beaverton, OR).

In Figure 1B, the optical emission spectra (OES) of micro DBD were measured by CCD spectrometry (HR400, Ocean Optics, Dunedin, FL). The intensity of the light emitted from the device was recorded in terms of wavelength (range from 280 to 407 nm).

### Cell culture

MC3T3 E1 from normal mouse osteoblast and SaOS-2 from osteosarcoma of mouse cell lines were obtained from the American Type Culture Collection (ATCC, Rockville, MD, USA), and maintained in a-MEM and RPMI 1640 with 10% heat inactivated fetal bovine serum (FBS) (Gibco), and 1% antibiotics and antimyotics (Gibco), respectively. The cells were plated in 35 mm culture dishes, and treated for differential times by 35 mm DBD NBP.

### Osteogenic differentiation

Osteogenic differentiation was induced according to established protocols. In brief, the cells were cultured to 80% confluency in basal media, and then changed to osteogenic induction media (OI) with 10% FBS, 10 mM β-glycerolphosphate, 50 μM L-ascorbic acid 2-phosphate, and 100 nM dexamethasone (Sigma-Aldrich). Von Kossa staining and Alizarin Red S (ARS) staining were performed for the visualization of calcium nodules. For von Kossa staining, cells were fixed with 4% paraformaldehyde overnight, and rinsed with distilled water. The cells were incubated several times with 1% silver nitrate solution under ultraviolet light for 1 hour, and rinsed with distilled water. To remove un-reacted silver, cells were washed with 5% sodium thiosulfate for 5 min. For ARS staining, cells were fixed with 4% paraformaldehyde for 10 min, stained with ARS solution for 1 hour in room temperature, then washed with PBS. The stained cells and mineral nodules were observed under phase contrast microscope. The ARS solution was resolved with 10% acetic acid, and read with microplate reader (Gen5) at 450 nm wavelength.

### Proliferation assay

NBP-treated cells were plated at 35 mm culture dishes in concentration of 1 × 10^5^ cells/dish at 37°C incubator. NBP-treated cells were evaluated by CCK-8 assay after 24 hours and 120 hours, respectively. The absorbance was measured at 450 nm by microplate reader (Gen5). Basal culture medium was used as a blank control.

### ALP activity

To analyze the effect of NBP on alkaline phosphatase (ALP) in both MC3T3-E1 and SaOS-2 cells, we measured ALP activity in the cell supernatants using colorimetric assay kit (BioVision, Milpitas Boulevard, Milpitas, CA, USA). ALP activity was measured at 405 nm in a micro plate reader (Gen5), and calculated with *p*NP standard curve.

ALP activity (U/ml) = A/V/T

A: amount of *p*NP generated by samples (μmol)

B: volume of sample added in the assay well (ml)

C: reaction time (min)

### Immunoblot assay

Immunoblotting was conducted according to our standard protocols, described previously [29]. The protein was extracted, quantified, and separated on SDS-PAGE gels, and electro-transferred to nitrocellulose membranes. The membranes were blocked in 3% BSA, and incubated with primary antibodies for FoxO1, p-FoxO1 (rabbit monoclonal, Cell Signaling Technology Inc., Danvers, MA, USA), PI3K (rabbit polyclonal, Cell Signaling), AKT, p-AKT, JNK, p-JNK, ERK, p-ERK, p38, p-p38 (rabbit monoclonal, Cell signaling) and β-actin (mouse monoclonal, Sigma-Aldrich, St Louis, MO). The blots were exposed to HRP-conjugated secondary rabbit IgG antibodies or mouse IgG antibodies, and analyzed by enhanced chemiluminescence (ECL) western blotting detection system (GE HealthCare Bio-Sciences, Piscataway, NJ, USA).

### Real time PCR analysis

Total RNA was purified from MC3T3-E1 and SaOS-2 cells after plasma treatment (RNeasy Kit, QIAGEN, AMBION, Inc., Texas, USA). For DNA synthesis, RNA of MC3T3-E1 and SaOS-2 was reverse-transcribed with specific primers: *PIK3CA, PIK3R1, PIK3R2, Runx2, osterix, ALP*, and *OCN* (Table 1) [30]. Two μl of each reverse transcription reaction product was used in the PCR reactions (iQ SYBR^®^ Green Supermix kit, Bio-Rad Laboratories, Richmond, CA). The amplifications were performed with the following protocol: 35 cycles of 95°C for 15 s, and 60°C for 60 s. The amplification curve was analyzed by delta-delta method.

∆∆Ct = (Cttarget – Ctreference) calibrator – (Cttarget – Ctreference) sample

**Table 1:** Primers for qRT-PCR

Gene	Forward primer	Reverse primer
*PIK3CA*	AGCCACACACTACATCAGTGGCT	ACAGGTCAATGGCTGCATCAT
*PIK3R1*	TGTCCGGGAGAGCAGTAAACA	CGCCGTCCACCACTACAGA
*PIK3R2*	AGCTGGACACACGGCTCCT	TGACAATCTGGTCCTGCTGGT
Osteogenic marker		
*Runx2*	AAATGCCTCCGCTGTTATGAA	GCTCCGGCCCACAAATCT
*Osterix*	AGCGACCACTTGAGCAAACAT	GCGGCTGATTGGCTTCTTCT
*ALP*	ATCTTTGGTCTGGCTCCCATG	TTTCCCGTTCACCGTCCAC
*Col1*	GGTCAAAGGTTTGGAAGCAG	TGTGAAATGCCACCTTTTGA
*OCN*	CCTGAGTCTGACAAAGCCTTCA	GCCGGAGTCTGTTCACTACCTT

### Flow cytometry

Assessment of double staining for FITC conjugated Annexin V and PI was performed according to the manufacturer's recommendation with Flow cytometry phenotyping. After plasma treatment, MC3T3-E1 cells and SaOS-2 cells were trypsinized, and incubated in cold binding buffer at 1 × 10^5^ cells/ml. The cells were stained with Annexin V/PI, and analyzed by flow cytometry (BD FACSCalibur, BD Biosciences, San Jose, CA, USA). The results were shown as mean percentage of positive cells and standard deviation of triplicate determinations.

### Statistical analysis

Statistical analysis was performed using Microsoft Excel analysis tools and SigmaPlot software. All data values were shown as mean ± standard deviation (SD). Comparison of all other results was performed by one-way analysis of variance (ANOVA) with Tukey's comparison analysis and the statistical significance was analyzed using Student *T* test and analysis of variance. Data was considered significantly different when *p < 0.05, **p < 0.01, ***p < 0.001. The Prism (Graphpad Software Inc,) and Excel Software (Microsoft Inc.) was used to compare groups.

## Abbreviations

NBP: non-thermal atmospheric pressure biocompatible plasma
RONS: reactive oxygen nitrogen species
DBD: dielectric barrier discharge
PI3K: phosphatidylinositol-3-kinases
AKT: serine/threonine-specific protein kinase
OCN: osteocalcin
Runx2: Runt-related transcription factor 2
COL1: collagen type 1
JNK: c-Jun N-terminal kinases
MST: mammalian Ste20-like kinases
